# Postpartum fever in the presence of a fibroid: *Sphingomonas paucimobilis* sepsis associated with pyomyoma

**DOI:** 10.1186/1471-2334-13-574

**Published:** 2013-12-05

**Authors:** Cosmo Del Borgo, Francesco Maneschi, Valeria Belvisi, Francesca Morelli, Angelo Vetica, Raffaella Marocco, Tiziana Tieghi, Miriam Lichtner, Claudio M Mastroianni

**Affiliations:** 1 Infectious Disease Unit, Fondazione Eleonora Lorillard Spencer Cenci, Sapienza University, Corso Della Repubblica 79, 04100 Latina, Italy; 2 Obstetrics and Gynecology Unit, SM Goretti Hospital, Latina, Italy

**Keywords:** Pyomyoma, Fever, *Sphingomonas paucimobilis* septicaemia

## Abstract

**Background:**

Pyomyoma is a life-threatening complication of uterine leiomyoma. It may occur in post- menopausal women, during pregnancy and in the postpartum period. Fever may be the only manifestation during the early stages of the disease. We detail the first reported case of postpartum pyomyoma-related sepsis due to *Sphingomonas paucimobilis,* a Gram-negative bacillus that is gaining recognition as an important human pathogen.

**Case presentation:**

A woman presented with an asymptomatic uterine fibroid and a two-week history of fever during the postpartum period. Suppurative uterine leiomyoma was diagnosed, and blood cultures grew *Sphingomonas paucimobilis.* The myoma was surgically removed from the uterus without hysterectomy. Intravenous antimicrobial therapy was given for fifteen days, and the patient was discharged from hospital in good condition.

**Conclusion:**

Pyomyoma should be considered in broad differential diagnosis of postpartum fever. This case highlights a unique disease manifestation of *S. paucimobilis*, an emerging opportunistic pathogen with increasing significance in the nosocomial setting.

## Background

Pyomyoma is a life-threatening complication of uterine leiomyoma [1]. This suppurative process is rare, especially in the postpartum period, in which it can develop insidiously. It can cause abdominal pain, but fever is generally the only symptom, especially in the early stages. As it is fatal without surgical treatment [2], thorough investigation of patients presenting with fever and leiomyoma is crucial, bearing in mind that radiological findings are often nonspecific.

Pyomyoma is usually associated with polymicrobial infection with microorganisms such as *Clostridium* sp, *Staphyloccocus sp*, and *Enterobacteriaceae*. Here, however, we report the case of a woman who, during the puerperal period, developed pyomyoma ascribed to *Sphingomonas paucimobilis* bacteraemia.

*Sphingomonas paucimobilis* is a yellow-pigmented, aerobic, non-fermenting, Gram-negative bacillus that thrives in a variety of natural environments such as soil and water [3,4]. It has also been detected in ultrapure water in industrial systems, and is able to form biofilms in water piping. Outbreaks associated with indwelling devices and contaminated fluid preparations have been described, and *S. paucimobilis* bacteraemia in various clinical settings has been reported with increasing frequency [5-8].

## Case presentation

In September 2012, a 37-year-old woman was hospitalized due to a two-week history of persistent fever. Thirty days before she had given birth to a healthy child through normal vaginal delivery in the 39^th^ week. The patient had a history of asymptomatic uterine fibroid, but no systematic follow-up had been performed, despite two first-trimester abortions in the two years prior to hospitalization. She had had no recent history of antibiotic treatment, and her records showed no previous invasive procedures, instrumentation or foreign bodies in the uterus.

On admission, body temperature was 39°C, blood pressure 105/50 mmHg, and pulse 100 beats/min. Her respiratory rate was 30 breaths/min. Physical findings were normal, except for tenderness in lower abdominal quadrants. Gynaecological examination revealed an enlarged uterus with tenderness upon deep palpation. Laboratory tests revealed a white blood cell count of 14,200/mm^3^ (81.5% neutrophils) and platelet count of 95,000/mm^3^. C-reactive protein and procalcitonin were 10.8 mg/dL and 59.71 ng/mL, respectively. Chest X-ray was negative for pleuroparenchymal lesions, and transthoracic echocardiogram was negative for valvular vegetations. Abdominal ultrasound revealed hepatosplenomegaly, but was negative for effusion and nodular lesions.

**Figure 1:**
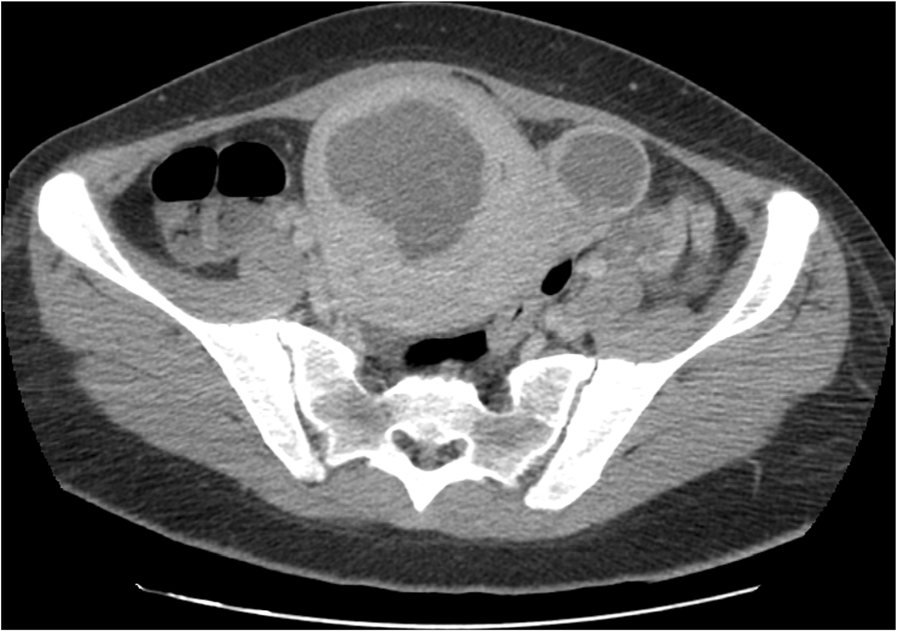
CT scan, abdominal view: mild bilateral hydroureteronephrosis and enlarged uterus with heterogeneous masses.

Since no evident source of infection was apparent, a full-body CT scan was performed. Images showed mild bilateral hydroureteronephrosis and an enlarged uterus with heterogeneous masses suggestive of leiomyoma (Figure 1).

The patient was accordingly started on meropenem and amikacin, but after a three-day course, her temperature remained high (39°C) and her clinical conditions failed to improve. Antibiotic treatment was therefore supplemented with teicoplanin and metronidazole.

Blood cultures grew *S. paucimobilis*, which was fully responsive to all tested antibiotics including β-lactams, carbapenems, and fluoroquinolones (Table 1).

**Table 1:** **Antimicrobial drug susceptibility of *Sphingomonas paucimobilis***

**Antimicrobial drug**	**Susceptibility**	**MIC (μg/ml)**
Ampicillin	S	<=2
Cefotaxime	S	<=1
Gentamicin	S	<=1
Amikacin	S	<=2
Levofloxacin	S	<=0.12
Meropenem	S	<=0.25
Cefepime	S	<=1
Tygeciclin	S	<=0.5

S, susceptible. MICs are based on EUCAST breakpoints.

A new CT scan documented a thickening of the pulmonary interstitium, bilateral basal pleural effusion, and slight effusion in the rectouterine pouch and pelvis. Transvaginal ultrasound confirmed uterine enlargement, and revealed two hypointense solid masses located over the fundal portion and left side of the uterine body.

**Figure 2:**
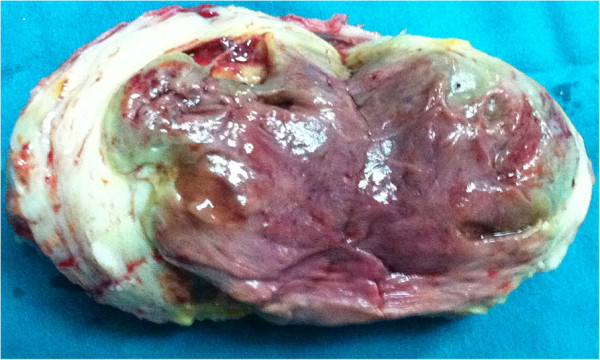
Surgical specimen: anterior view of 10 cm dissected pyomioma of uterine fundus.

Explorative laparotomy revealed a large myoma (10 cm) located over the fundus of the enlarged uterus, and a smaller myoma (3 cm) on the left side of the uterine body. A small amount of ascitic fluid was also present in the rectouterine pouch. The fundal myoma was dissected from the uterus, resulting in an outflow of purulent fluid and colliquative necrotic material (Figure 2). Hysterectomy was not deemed necessary, and no complications arose during the postoperative course.

Antibiotic therapy with teicoplanin, metronidazole and meropenem was given for fifteen days, and histopathology confirmed the diagnosis of pyomyoma. Aerobic and anaerobic cultures obtained from the specimen did not yield bacterial growth. The patient was discharged from hospital in good condition on postoperative day 16.

## Conclusions

Pyomyoma (also known as suppurative leiomyoma) is a rare but life-threatening condition resulting from infarction and infection of uterine leiomyoma [1,2]. Predisposing risk factors include gynaecological surgery, uterine instrumentation, vascular insufficiency, and immunodeficiency. Pyomyoma may occur in both post-menopausal and pre-menopausal women, but the risk of suppurative complications of myoma is increased by pregnancy.

Nonetheless, since 1945, only 17 pyomyoma cases (including our patient) arising during pregnancy or the postpartum period have been reported (Table 2). The previous case reports indicate that infection may arise as consequence of abortion, caesarean section or intravenous drug abuse, and most infections recorded in the postpartum period developed insidiously over days to weeks between delivery and onset of symptoms.

**Table 2:** Pyomyoma during pregnancy or in postpartum period

**Author and year**	**Onset of symptoms**	**Underlying condition**	**Surgical intervention**	**Pathogen**	**Outcome**
Dubois [9]	3 weeks postpartum	-	vaginal expulsion of pyomyoma	N/A	N/A
Ruch [10]	5 month pregnancy/1 day postpartum	-	total abdominal hysterectomy + appendectomy	Gram positive cocci	N/A
Wong [11]	3 days post-abortion	18 weeks IUD associated spontaneous abortion	total abdominal hysterectomy + left salpingectomy	*Staphylococcus aureus + Serratia marcescens*	N/A
Prichard [12]	9 weeks post-abortion (spontaneous)	-	total abdominal hysterectomy + bilateral salpingectomy	*Streptococcus milleri*	N/A
Tobias [13]	10 weeks post-abortion	uterine fibroids, 15 weeks elective abortion	total abdominal hysterectomy + bilateral salpingectomy	*Enterococcus faecalis*	S
Prahlow [14]	12 weeks pregnancy	intravenous drug abuse	total abdominal hysterectomy + bilateral salpingectomy + partial omentectomy	*Staphylococcus aureus*	N/A
Grüne [15]	25 weeks pregnancy	-	cesarean section + myomectomy	*Klebsiella pneumoniae*	S
Lin [16]	6 days postpartum	uterine fibroids, preterm premature rupture of membranes, surgical site infection (cesarean section)	total abdominal hysterectomy	*Escherichia coli + Candida parapsilosis*	S
Karcaaltincaba [17]	7 days post-abortion (spontaneous)	-	myomectomy	*Peptostreptococcus tetradus*	S
Mason [1]	21 days postpartum	manual removal of adherent placenta after full term vaginal delivery	myomectomy	no growth	S
Nguyen [18]	3 weeks postpartum	41 weeks cesarean section delivery, severe chorioamnionitis	total abdominal hysterectomy	*Escherichia coli*	S
Laubach [19]	18 hours post- abortion (spontaneous)	-	vaginal expulsion of pyomyoma + computed tomography- uided drainage	*Escherichia coli*	S
Laubach [19]	postpartum period	33 weeks cesarean section delivery, preterm premature rupture of membranes, chorioamnionitis	computed tomography-guided drainage	*Escherichia coli + Candida albicans + Candida dubliniensis*	S
Laubach [19]	N/A	29 weeks cesarean section delivery, preterm premature rupture of membranes, surgical site infection	computed tomography-guided drainage + subtotal abdominal hysterectomy	*Enterococcus faecalis + Streptococcus spp.*	S
Shaaban [20]	8 weeks postpartum	cesarean section	myomectomy	*Staphylococcus lugdunensis*	S
Kobayashi [2]	20 weeks pregnancy	-	myomectomy (21 weeks of gestation)	Anaerobic Gram-negative rods	S
Present case, 2013	30 days postpartum	uterine fibroids	myomectomy	*Sphingomonas paucimobilis*	S

*Abbreviations*: *N/A* not available, *IUD* intra uterine device, *S* survived.

Abdominal pain was reported in some cases, but fever was generally the only symptom, especially in the early stages. Hence, attending physicians need to be particularly vigilant in cases of postpartum fever and leiomyoma, as neither radiology nor pelvic ultrasound are likely to be diagnostic. Indeed, although pyomyoma has been associated with poor obstetric outcomes, prompt surgical intervention can improve clinical results [2].

In post-menopausal women and patients with small multiple intramural lesions or ruptured myomas, total abdominal hysterectomy is the preferred option. Nevertheless, a more conservative approach based on myomectomy or computed tomography-guided drainage of pyomyoma is now feasible [19], and indeed preferable in pre-menopausal women, in order to preserve their fertility. Myomectomy can even be safely performed during the first and second trimesters of pregnancy, but in all cases the prompt initiation of broad-spectrum antibiotic treatment is critical for good outcome.

Pyomyoma is usually associated with polymicrobial infection [21] by microrganisms such as *Clostridium* sp, *Staphyloccocus sp*, *Streptococcus sp.*, *Proteus* sp, *Serratia marcescens*, *Enterococcus faecalis*, *Klebsiella pneumoniae*, *Escherichia coli and Candida* sp. This, however is the first report of septicaemia linked to pyomyoma in the puerperal period caused by *S. paucimobilis*.

*S. paucimobilis*, a non-fermenting Gram-negative bacillus, is widely distributed in nature and hospital environments. It has been implicated in a variety of human infections, including bacteraemia, pneumonia, catheter-related infections, meningitis, peritonitis, osteomyelitis, septic arthritis, postoperative endophthalmitis, lung empyema, splenic abscesses, and urinary tract infections [22]. The most common comorbidities included malignancy, immunodeficiency, and diabetes mellitus.

Although sporadic or community-acquired infections have been described, this organism is increasingly associated with nosocomial settings, and hospital-acquired bacteraemia accounts for two-third of reported cases. Nosocomial outbreaks are usually related to manipulation of indwelling devices or contamination of sterile fluids [23], and outbreaks are more likely to occur in dialysis units [24,25], haematology and oncology wards [5,6], and neonatal intensive care units [7].

Kriet et al. reported a case of endogenous postpartum panophthalmitis induced by *S. paucimobilis* in a primiparous 39-year-old with a diagnosis of postpartum endomyometritis [26], presumably contracted in the hospital setting. Likewise, in our case, although there was no clear evidence of nosocomial risk factors, such as previous antimicrobial therapy, invasive procedures, or placement of foreign bodies, and no recent cases of *S. paucimobilis* bacteraemia in our hospital, we cannot rule out bacterial colonization of the hospital water system.

In conclusion, this case highlights a singular manifestation of infection by *S. paucimobilis*, an opportunistic pathogen emergent in the nosocomial setting. Hence its potential implication in unusual life-threatening infections, such as pyomyoma, should be kept in mind. Furthermore, pyomyoma should be considered in the broad differential diagnosis of postpartum fever, especially if concomitant with bacteraemia and uterine fibroids but no other obvious source of infection. Indeed, this suppurative process is invariably fatal without surgery [27] combined with appropriate antibiotic treatment. Nowadays, however, surgery does not necessarily involve hysterectomy, and potential fertility can be preserved by means of conservative procedures such as myomectomy [19].

## Consent

Written informed consent has been obtained from the patient for publication of this Case report. A copy of the written consent is available for check by the Editor of BMC Infectious Diseases, if needed.

## Competing interests

The authors declare that they have no competing interests.

## Authors’ contributions

CDB collected the clinical data and drafted of the manuscript, VB, FM, AV, and ML managed the patient, and supported CDB in the collection of clinical data and drafting of the manuscript, RM and TT performed the literature search and support CDB in the drafting of and revising the manuscript, FM contributed to the clinical and therapeutic management from a gynecologic point-of-view, and revised the gynecologic details of the manuscript, CMM supervised the clinical case interpretation, participated in the coordination and concept of the manuscript, and helped with the draft of the manuscript. All authors read and approved the manuscript.

## Pre-publication history

The pre-publication history for this paper can be accessed here:

http://www.biomedcentral.com/1471-2334/13/574/prepub
